# Effectiveness of different acupuncture therapies for chronic cancer pain: A protocol for systematic review and Bayesian network meta-analysis

**DOI:** 10.1097/MD.0000000000027965

**Published:** 2022-01-28

**Authors:** Qingyun Wan, Hao Chen, Xiaoqiu Wang, Hanqing Xi, Shiyu Zheng, Shuting Luo, Wenzhong Wu, Rui Pan

**Affiliations:** a Jiangsu Province Hospital of Chinese Medicine, Affiliated Hospital of Nanjing University of Chinese Medicine, Nanjing, China; b Nanjing University of Chinese Medicine, Nanjing, China; c Jiangsu Cancer Hospital, Affiliated Cancer Hospital of Nanjing Medical University, Nanjing, China.

**Keywords:** a systematic review, acupuncture, chronic cancer pain, network meta-analysis

## Abstract

**Background:**

Pain is a common and distressing symptom experienced by cancer patients. Previous research found acupuncture was associated with significant reductions in pain intensity and opioid use. Acupuncture therapies are various, and the difference in efficacy and safety has never been assessed. This paper aims to assess and rank the effectiveness of the different acupuncture methods and provide an acupuncture treatment guideline for relieving chronic pain in cancer survivors.

**Methods:**

Four English databases (PubMed, Embase, Cochrane library, and Web of Science) and 4 Chinese databases (China National Knowledge Infrastructure, Wanfang Data, and Chinese Biomedical Literature Database) will be searched for randomized controlled trials (RCTs) published from the database inception to November 30, 2021. The primary outcomes will be patient-reported pain intensity measured by the Brief Pain Inventory, Visual Analog Scale, Verbal Rating Scale, Numerical Rating Scale, and other valid outcome measures. The Grading of Recommendations Assessment, and Development and Evaluation System will evaluate the quality of evidence. Bayesian network meta-analysis will be performed in WinBUGS V.1.4.3 to determine the comparative effectiveness of the acupuncture therapies.

**Results:**

This study will quantify the effectiveness of each acupuncture intervention for chronic cancer pain with pain scores and the use of analgesics. The adverse events of acupuncture treatment for cancer pain will also be reported.

**Conclusion:**

The conclusion of our study will help physicians and patients choose suitable acupuncture methods to manage cancer pain.

## 1 Introduction

### 1.1 Description of the condition

The number of cancer survivors was up to 15.5 million in 2016 in the United States and is expected to reach 26.1 million by 2040 with the aging population and advances in early diagnosis and treatment methods.^[1]^ Pain, one of the most common and troublesome symptoms affecting patients with cancer, is experienced by 39% of cancer survivors, but inadequately controlled in nearly half of them.^[2,3]^ Poor pain control is associated with impaired quality of life, lower adherence to treatment, and higher health care costs.^[1,4]^

Opioid analgesics are regarded as a gold standard in cancer-related pain, but clinicians must carefully assess whether their benefits counterbalance potential complications.^[5]^ Opioid addiction among cancer survivors has been estimated to be as high as 7.7%.^[6]^ Hundreds of thousands of individuals in the United States have died of opioid-related causes, millions have become addicted, and billions of dollars of economic value have been spent.^[7]^ The ongoing opioid crisis in the United States has triggered new skepticism about opioid use and difficulties in cancer pain management.^[8,9]^ Besides, nonpharmacologic methods are recommended for the treatment of adult cancer pain according to the NCCN Clinical Practice Guidelines in Oncology (NCCN Guidelines).^[10]^

### 1.2 Description of the intervention

Acupuncture, as one of the non-pharmacologic methods, has been successfully applied in the cure or relief of 64 different symptoms including pain,^[11,12]^ and is available in over 78 countries, according to the World Health Organization (WHO, 2003).^[13]^ Clinical evidence has demonstrated clinically significant relief of cancer pain and reduced use of analgesics by adopting acupuncture.^[14,15]^ More than 20 systematic reviews have investigated the association of acupuncture with cancer pain; however, substantial heterogeneity lowered the level of certainty of the evidence.^[15]^ The diversity of acupuncture therapies is likely a factor contributing to substantial heterogeneity.

Acupuncture therapies for cancer pain were shown as follows: Monotherapy, including manual acupuncture (MA), electro-acupuncture (EA), auricular acupuncture (AA); Combination therapy such as acupuncture with analgesics, manual acupuncture with auricular acupuncture, acupuncture with moxibustion.^[15–18]^ Different acupuncture interventions will be included in the systematic review and network meta-analysis.

### 1.3 Objective

This study aims to assess and rank the effectiveness of different acupuncture therapies and provide a prioritized acupuncture-based treatment regimen for relieving chronic pain in cancer survivors by Bayesian network meta-analysis. Randomized clinical trials that compared acupuncture with sham control, analgesic therapy, or usual care for managing cancer pain will be included.

## 2 Methods

This protocol conforms to the Preferred Reporting Items for Systematic Reviews and Meta-Analysis Protocols (PRISMA-P) statement,^[19]^ and has been registered in PROSPERO (CRD42020207158).

### 2.1 Eligibility criteria

#### 2.1.1 Types of studies

All randomized controlled trials (RCTs) comparing acupuncture therapies with analgesics interventions, placebo, or no intervention for patients with cancer-related pain will be included without language or region restrictions. Studies with unavailable data will be excluded.

#### 2.1.2 Types of participants

Patients (older than 18 years) who were diagnosed with cancer reported pain.^[20]^ Pain resulting from the development of cancer self and/or cancer treatments will be included, but breakthrough or acute pain are excluded.

#### 2.1.3 Types of intervention

Eligible interventions will be manual acupuncture, electro-acupuncture, auricular acupuncture, moxibustion, or a combination of these, regardless of acupoint selection, acupuncture manipulations, or treatment course. The control group can be sham acupuncture, analgesics, or usual care managing cancer pain. Trials comparing 2 acupoint selections (e.g., scalp acupuncture vs body acupuncture) or acupuncture manipulations (e.g., electroacupuncture vs manual acupuncture) will be excluded.

#### 2.1.4 Types of outcome measure

##### 2.1.4.1 Primary outcomes

Patient-reported pain intensity or pain relief measured by Visual Analog Scale (VAS), Verbal Rating Scale (VRS), the Brief Pain Inventory, Numerical Rating Scale (NRS), and other validated instruments. Results measured by different scales will be converted to the corresponding grade for data integration (0 points indicating no pain, and 10 points indicating most severe pain). Besides, pain lasting time after the intervention will be reported and included in the meta-analysis.

##### 2.1.4.2 Secondary outcomes

Secondary outcomes will include Pain improvement percentage measured by valid scales such as VAS was calculated as (the pain score before treatment - the pain score after treatment)/ the pain score before treatment; Quality of life indicated by scales European Organization for Research and Treatment of Cancer Quality of Life Questionnaire^[19]^; Consumption of analgesics or changes in concurrent treatments; and Adverse events of interventions.

### 2.2 Search strategy

Four English databases (PubMed, Embase, Cochrane library, and web of science) and 4 Chinese databases (China National Knowledge Infrastructure, Wanfang Data for Chinese Technical Periodicals, and Chinese Biomedical Literature Database) will be searched for RCTs published from the database inception to November 30, 2021. Date search comprised 3 components: clinical condition (i.e., cancer, tumor, neoplasm, carcinoma, pain, analgesia), intervention (i.e., acupuncture, manual acupuncture, electroacupuncture, auricular acupoint acupressure), study type (RCT). The search strategy of the PubMed database is summarized in Table 1. In case of missing other eligible studies, reference lists of relevant publications, including trials, reviews, and meta-analysis, will be reviewed for a manual search.

**Table 1:** Search strategy used for the PubMed database.

Number	Search items
#1	Neoplasms [Mesh] OR Neoplasia [Title/Abstract] OR Tumor [Title/Abstract] OR Cancer [Title/Abstract] OR Malignancy [Title/Abstract] OR Malignant Neoplasm [Title/Abstract] OR Benign Neoplasm [Title/Abstract]
#2	Pain [Mesh] OR analgesia [Mesh]
#3	#1 AND #2
#4	Acupuncture [Mesh] OR Acupuncture therapy [Mesh] OR Acupuncture Treatment [Title/Abstract] OR Acupuncture, Ear [Mesh] OR Auricular Acupuncture [Title/Abstract] OR Acupuncture points [Mesh] OR Acupoints [Title/Abstract]
#5	#3 AND #4
#6	Randomized Controlled Trials as topic [Mesh] OR Clinical Trials, Randomized [Title/Abstract] OR Controlled Clinical Trials, Randomized[Title/Abstract] OR Randomized Controlled Trial [Publication Type] OR Intention to Treat Analysis [Mesh] OR Controlled Clinical Trials as Topic [Mesh]
#7	#5 AND #6

### 2.3 Study selection and data extraction

**Figure 1:**
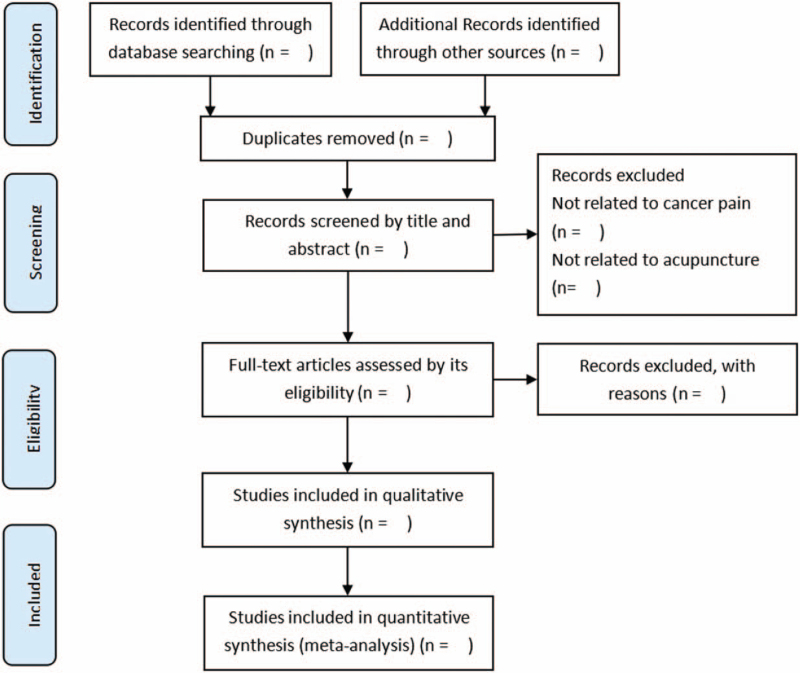
PRISMA flow diagram.

All study selection will be independently performed by 2 reviewers (SL and HX) using a predetermined protocol according to the PRISMA flow diagram (Fig. 1).^[19]^ Divergences between 2 reviewers will be solved by negotiating with a third reviewer (XW). Data extraction will be based on a standardized data form, including Trial characteristics (author, publication year, study design, location); Patient characteristics (sample size, age, gender ratio, cancer type, cancer treatment method, treatment status when receiving acupuncture); Details of intervention and control (form, acupoints, frequency and treatment duration); and Data of outcomes referred above.

### 2.4 Quality assessment

The quality of RCTs will be assessed with the Cochrane Collaboration risk of bias tool including 6 domains.^[21]^ Six specific domains (sequence generation, allocation concealment, blinding of participants and outcome assessment, incomplete outcome data, selective outcome reporting, and other potential threats), for each study, will be assigned a risk of bias (low, high, or unclear). If there are any missing data, corresponding authors will be contacted and asked to provide relevant details when necessary.

The level of the quality of evidence for main outcomes will be appraised with the Grading of Recommendations Assessment, Development, and Evaluation (GRADE) system approach, including study limitations, inconsistency, indirectness, imprecision, and publication bias.^[22]^ All Quality assessments procedure will be performed independently by 2 reviewers (SL and HX), and a third reviewer (XW) will join to resolve by consensus if a discrepancy occurs.

### 2.5 Statistical analysis

#### 2.5.1 Pairwise meta-analysis

The characteristics about participants, intervention, comparisons of the included RCTs will be summarized in the table. Continuous data will be performed by calculating the effect size and 95% confidence interval (95% CI) with a random-effects model, and dichotomous data will be computed with risk ratios. Heterogeneity among trials will be identified by the *χ*^*2*^ test and reported as *I*^*2*^. STATA version 15.1 software (Stata Corporation, College Station, TX) will be used for statistical analysis. Two-sided *P* < .05 is regarded as statistical significance.

#### 2.5.2 Network meta-analysis

The network meta-analysis will be conducted with a Bayesian hierarchical random-effects model using WinBUGS (version 1.4.3; MRC Biostatistics Unit, Cambridge, UK) to combine and compare direct and indirect evidence of interventions for cancer pain. Inconsistency will be analyzed by both design-by-treatment and loop-specific approaches using a node-splitting test.^[23,24]^ The effect size for the continuous data will be calculated as a mean difference with 95% CIs, while dichotomous outcomes will be presented as risk ratios with 95% CIs. The surface under the cumulative ranking curve (SUCRA) will assess superiority probabilities of efficacy and safety outcomes for each intervention, and presented as percentages.^[25]^ Higher SUCRA values indicate better effects or safer intervention for cancer pain.

#### 2.5.3 Subgroup analysis

In case of possible substantial heterogeneity, we will explore the possible sources using network meta-regression and subgroup analysis. Subgroup analysis will be stratified according to likely factors, including patients’ characteristics (i.e., age, gender), interventions, control group.

#### 2.5.4 Sensitivity analysis

Sensitivity analysis will be performed to get a definitive conclusion of the review. Trials with missing data, small sample size, or rated as high risk of bias for methodological quality will be re-considered.^[26]^

#### 2.5.5 Assessment of publication bias

Publication bias of the included studies will be assessed by funnel plots and Egger's test for asymmetry of primary outcomes.^[27]^

## 3 Discussion

This study is expected to provide a ranking of acupuncture interventions for chronic cancer pain, regarding efficacy and safety by network meta-analysis. The results will help clinicians and patients choose preferred acupuncture therapy in the treatment of cancer pain. And we hope to provide evidence for policymakers to include effective and safe acupuncture therapies in the management of cancer.

## Author contributions

**Conceptualization:** Qingyun Wan, Wenzhong Wu, Rui Pan

**Data curation:** Xiaoqiu Wang, Hanqing Xi, Shuting Luo

**Formal analysis:** Qingyun Wan, Hao Chen

**Project administration:** Xiaoqiu Wang, Hanqing Xi, Shiyu Zheng

**Supervision:** Qingyun Wan, Hao Chen, Wenzhong Wu, Rui Pan

**Writing – review & editing:** Qingyun Wan, Hao Chen

**Conceptualization:** Qingyun Wan, Wenzhong Wu, Rui Pan.

**Data curation:** Xiaoqiu Wang, Hanqing Xi, Shuting Luo.

**Formal analysis:** Qingyun Wan, Hao Chen.

**Project administration:** Xiaoqiu Wang, Hanqing Xi, Shiyu Zheng.

**Supervision:** Qingyun Wan, Hao Chen, Wenzhong Wu, Rui Pan.

**Writing – review & editing:** Qingyun Wan, Hao Chen.
